# The value of Serum BNP for diagnosis of intracranial injury in minor head trauma

**DOI:** 10.1186/1749-7922-9-16

**Published:** 2014-02-10

**Authors:** Ali Demir, Cemil Kavalci, Muhittin Serkan Yilmaz, Fevzi Yilmaz, Tamer Durdu, Mehmet Ali Ceyhan, Fatih Alagoz, Cihat Yel

**Affiliations:** 1 Emergency Department, Numune Training and Research Hospital, Ankara 06370, Turkey; 2 Emergency Department, Baskent University Faculty of Medicine, Ankara 06450, Turkey

**Keywords:** Emergency, Head trauma, Brain natriuretic peptide

## Abstract

**Objective:**

Head injury is the main cause of death among individuals younger than 45 years old. Cranial Computerized tomography (CT) is commonly used for diagnosis of head injury. Brain Natriuretic Peptide (BNP) is a peptide originally isolated from brain ventricles. The main aim of this study is to investigate BNP as an indicator of head injury among patients presenting to emergency department (ED) with minor head trauma.

**Methods:**

This was a prospective study conducted at the emergency department of the Numune Training and Research Hospital. A total of 162 patients who presented to the ED with minor head injury were enrolled. The patients were categorized into 2 groups as the cranial CT-negative and positive groups. The normality of the data was tested using One Sample Kolmogorov Smirnov test. Mann–Whitney U test was used to compare 2 independent groups while the Kruskal-Wallis test was utilized for comparison of more than 2 groups. A p-value of <0.05 was considered to be significant.

**Results:**

Ninety-six (59.3%) patients were male and 66 (40.7%) were female. The cranial CT-negative group had a median BNP level of 14.5 pg/ml while the cranial CT-positive group had a median BNP level of 13 pg/ml. There was no statistically significant difference between these two groups for serum BNP levels (p > 0.05).

**Conclusion:**

This study suggested that serum BNP level wasn’t used in defined of intracranial injury.

## Introduction

Head traumas and traumatic cerebral injuries constitute a major etiological factor for mortality and long-term morbidity especially in adolescents, young adulthood, and elderly [1]. Motor vehicle accidents, falls from a height, assaults, and gunshot injuries are the most common causes of head injuries. Of all head injuries, 80% are minor, 10% are moderate, and 10% are major injuries [1,2]. Cranial Computerized tomography (CT) is often ordered during emergency management of patients with head trauma. Unfortunately, CT is an expensive examination, not available in everywhere and puts patients at risk for long-term risks of radiation.

Previous studies have reported that some serum markers including neuron specific enolase (NSE), S100b, Tau protein, and malonyl dialdehyde (MDA) are increased in head trauma patients [3-6]. BNP, a natriuretic peptide consisting of 32 amino acids, is an important biomarker in establishing cardiovascular disorders including congestive heart failure and ischemic cardiomyopathy. It is commonly used both for determination of presence and degree of left ventricular systolic and diastolic dysfunction. It is also a predictor of prognosis after myocardial infarction [7,8]. In addition to its cardiovascular applications, some previous reports have also suggested that it can be used in head trauma [7-13].

The present study aimed to investigate whether BNP measurement can establish head injury in patients presenting to the emergency department with minor head trauma. If the answer is yes, excess CTs could be avoided which will reduce unnecessary costs and patients’ radiation exposure.

## Materials and method

This was a prospective, case–control study conducted at the emergency department of the Numune Training and Research Hospital. It included a total of 162 patients with head trauma admitting to the emergency department who met the study inclusion criteria. The inclusion and exclusion criteria are listed on Table 1.

**Table 1:** The criteria for inclusion or exclusion of patients to the study

**Criteria for inclusion to the study**	**Criteria for exclusion from the study**
To be admitted to the emergency department because of a head trauma.	To be younger than 18 years old.
To be older than 18 years old.	To refuse to participate the study.
To give his/her consent to participate in study.	Having a known neurological disease.
	Having a known cardiac insufficiency.

Demographic features of the study participants, trauma mechanisms, concurrent injuries, time elapsed after trauma, GCS scores, findings on physical examination, cranial CT results were also recorded. Trauma severity was assessed using GCS.

The study population was grouped into 2 groups as cranial CT-negative group (Group 1) that had normal head CT findings and linear fracture, and cranial CT-positive group (Group 2) that had intracranial abnormalities including brain edema, epidural or subdural hematoma, subarachnoid or intraparenchymal hemorrhage, cerebral contusion, or a depressed skull fracture. Cranial CT reports were retrieved from the hospital automation system.

The study patients underwent a head CT as necessary and serum BNP measurement with Abbot Architect kit (normal range of 0–100 pg/ml) at admission.

Clinical and demographic features of the patients were stored in a computer database. Serum BNP levels were compared between both groups. Statistical analyses were performed using SPSS 15.0 software package. Mean ± SD, median, interquartile range, and percentage values were calculated for demographic and clinical features of the study participants. Median and interquartile range values were calculated for BNP levels. Categorical variables were compared with χ^2^ test. The normality of the study data was tested by means of One Sample Kolmogorov Smirnov test. As a result of the analysis, non-parametric tests were used in the analysis. As such, Mann–Whitney U test was used for comparison of two independent continuous groups, while Kruskal-Wallis test was used for multiple continuous groups. Spearman’s test used to investigation a association between Serum BNP levels and elapsed time after the event. A significance level of *p* < 0.05 was accepted for all statistical tests.

### Approval by the ethics committee

This study was approved by the Local Ethics Committee of the Numune Training and Research Hospital.

## Results

The present study was completed at the ED of the Numune Training and Research Hospital during summer months of 2013. A total of 162 patients meeting the inclusion criteria were enrolled. Group 1 and 2 included 148 and 14 patients, respectively.

### Demographic and clinical data

Ninety-six (59.3%) patients were male and 66 (40.7%) were female. Demographic and clinical findings are showed in Table 2.

**Table 2:** Demographic characteristics of the patients

	**Group 1**	**Group 2**	***p***
**Age (average, years)**	**49.18 ± 20.5**	**42.93 ± 22.1**	p > 0.05*
Gender (n)			
Male	84	12	p < 0.05**
Female	64	2	
Trauma mechanism (n)			
Motor vehicle accident	32	1	p > 0.05**
Pedestrian	9	1	
Falling	61	7	
Violent assaults	46	5	
Accompanying trauma	9	3	p < 0.05**
Bnp levels (median, IQR) (pg/ml)	14.5 (33)	13 (139)	p > 0.05*

*Mann-Witney U test, ** χ^2^ test.

The most common symptoms were headache (87%), vomiting (13%), amnesia (3.7%), unconsciousness (5%), and somnolence (3%). The most common signs on physical examination were scalp laceration (44.4%), scalp hematoma (38.8%), and raccoon eye (0.6%). Findings of head CT are given on Table 3. One hundred and thirty-four (82.7%) patients were discharged from the hospital and 28 (17.3%) were hospitalized.

**Table 3:** Cranial CT findings of the patients

**Finding**	**Number (n)**	***Percentage (%)***	**GCS (n) (14/15)**
Normal	146	*90.1*	8/138
Linear fracture	1	*0.6*	0/1
Cerebral edema	1	*0.6*	0/1
Subarachnoid hemorrhage	4	*2.5*	0/4
Compression fracture	2	*1.2*	0/2
Parenchymal haemorrhage	1	*0.6*	0/1
Contusio cerebri	2	*1.2*	0/2

### BNP

Median serum BNP level was 14.5 (33) pg/ml in Group 1 and 13 (139) pg/ml in Group 2. There was no not significantly different with respect to median BNP levels between two groups (p > 0.05). Median BNP level was 10 (21) pg/ml in males and 28.50 (56) pg/ml in females. There was a significant difference between both genders with regard to median BNP levels (Z = −4.29, p < 0.05).

The patients were divided in to 2 groups. Group 1 consisted of patients with admitted to our department within 0–12 hours after events whereas group 2 consisted of patients with admitted to our department within 13–24 hours after events. There was a no significant difference between both two groups with regard to median BNP levels (Z = −1.52, p > 0.05). There was no correlation between serum BNP levels and elapsed time after the event (r = 0.125, p > 0.05). Serum BNP levels according to trauma severity are given on Table 4. There was no correlation between serum BNP levels and trauma severity (r = −0.037, p > 0.05).

**Table 4:** BNP levels by various trauma mechanism and trauma severity

**Trauma mechanism**	**BNP (pg/ml)**	**p value**
Motor vehicle accident	15 (25)	p > 0.05*
Pedestrian	10 (53)	
Falling	22.5(56)	
Violent assaults	11(22)	
Glasgow coma score		
14 (n = 8)	10.5(27)	p > 0.05**
15 (n = 154)	14.5(34)	

*Kruskall-Wallis test, **Mann–Whitney U test.

Our patients in group 2 were hospitalized in neurosurgery service. They were discharged after treatment.

## Discussion

Only a few studies have reported increased serum BNP levels in patients with head trauma [7-10]. Costa et al. reported that serum BNP levels did not increase in patients with head injury and it had no correlation with cerebral salt-wasting syndrome [12]. Kavalci et al. reported that serum BNP might be useful in evaluation of head trauma [13]. Cevik et al. demonstrated that BNP levels exceeding 10 pg/ml were associated with an intracranial abnormality in patients with head injury [7]. Sviri et al. showed that serum BNP levels increased immediately following head injury [8]. Similarly, Lu et al. reported that BNP levels increased in patients with head trauma [9]. Cevik et al. showed that serum BNP levels significantly differed between patients with and without head trauma [7]. In contrast, we did not detect any significant difference between the 2 groups. We believe that this resulted from a low patient number in Group 2. We suggest that further studies with larger sample size may establish a relationship between serum BNP and head trauma.

Neither, Çevik et al. nor Kavalci et al. showed a significant correlation between trauma mechanism and serum BNP. We also found a similar result. BNP appears to be released into bloodstream in all kinds of head trauma.

Çevik et al. reported a significant relevance between delay in admission and BNP levels. They showed that a positive correlation exists between admission time and BNP levels [7]. Kavalci et al. showed that there was no significant correlation between the serum BNP levels and admission time [13]. Our results are support to Kavalci et al.

GCS is commonly used for assessment of neurological status of head trauma patients. There is a general agreement on the predictive power of GCS in patients with mild and serious head trauma, although there are various approach considerations with respect to radiological evaluation of minor head trauma cases. Thus, studies aiming to establish the indications of CT scanning of the head region or criteria for hospital admission by using some biochemical markers and clinical features [3-5]. Some reports suggested that the severity of head trauma and serum BNP levels are not significantly correlated [7,10,13]. Wu et al., in contrast, reported that serum BNP levels increased to a greater extent in patients with more severe head trauma [11]. We found no significant correlation between head trauma severity and serum BNP levels. However, all of our patients with minor head trauma group. This subject should be further clarified with adequate studies.

In a study by Çevik et al. serum BNP levels were significantly higher in patients with an intracranial lesion compared to those who did not. Cevik et al. proposed that serum BNP levels can be used as a surrogate marker of head trauma [7]. In contrast, Stewart et al. and Kavalci et al. suggested that this biomarker has no any appreciable value for this indication [10]. Since our results were in line with equality of BNP elevation in both patient groups, they support the results of the studies conducted by Stewart et al. and Kavalci et al.

## Conclusion

Our results suggested that serum BNP was not an adequate marker for determination of an intracranial pathology in patients with minor head trauma. As to date conflicting results have been reported, further studies with larger sample size should be followed in order to establish a possible link between serum BNP and minor head trauma.

### Limitation of the study

Since the number of patients in the present study is too low, the power of the study fell short to draw any meaningful conclusion. Moreover, the patient number in Group 2 was even lower (14 patients). Despite these limitations, our study demonstrated that there was no significant difference between Group 1 and 2 although all patients in the study had demonstrable intracranial lesions. Another limitation, We didn’t perform a serial BNP measurements because it is expensive.

## Competing interests

The authors declare that they have no competing interests.

## Authors’ contributions

The quantitative analysis was planned by CK, EDA, AD. Study data were analyzed by CK and interpreted by FY, MAC. The first version of the manuscript was drafted by AD, MSY, BMS. All authors contributed to the edition and revision of the manuscript and the final version of the article was reviewed and approved by all authors.
